# Performance Evaluation of Attribute-Based Encryption in Automotive Embedded Platform for Secure Software Over-The-Air Update

**DOI:** 10.3390/s21020515

**Published:** 2021-01-13

**Authors:** Michele La Manna, Luigi Treccozzi, Pericle Perazzo, Sergio Saponara, Gianluca Dini

**Affiliations:** 1 Department of Information Engineering (DII), University of Pisa, 56122 Pisa, Italy; l.treccozzi@studenti.unipi.it (L.T.); pericle.perazzo@unipi.it (P.P.); Sergio.saponara@unipi.it (S.S.); Gianluca.dini@unipi.it (G.D.); 2 Department of Information Engineering (DINFO), University of Florence, 50121 Florence, Italy

**Keywords:** Attribute-Based Encryption (ABE), over the air update (OTA), automotive gateways

## Abstract

This paper aims to show that it is possible to improve security for over the air update functionalities in an automotive scenario through the use of a cryptographic scheme, called “Attribute-Based-Encryption” (ABE), which grants confidentiality to the software/firmware update done Over The Air (OTA). We demonstrate that ABE is seamlessly integrable into the state of the art solutions regarding the OTA update by showing that the overhead of the ABE integration in terms of computation time and its storage is negligible w.r.t. the other overheads that are introduced by the OTA process, also proving that security can be enhanced with a minimum cost. In order to support our claim, we report the experimental results of an implementation of the proposed ABE OTA technique on a Xilinx ZCU102 evaluation board, which is an automotive-oriented HW/SW platform that is equipped with a Zynq UltraScale+ MPSoC chip that is representative of the computing capability of real automotive Electronic Control Units (ECUs).

## 1. Introduction

Over the last few decades, we saw a complete transformation of the automotive world. Vehicles increasingly rely on electronic components to provide new features to the customers. With well over 80 ECU’s per vehicle [1], software maintenance is a serious issue. In industry, it has been estimated that the number of bugs per 1000 lines of code oscillates from 0.5 to 25 [2]. It would be foolish to think that a vehicle that is on the market has no bugs, and it would be even more foolish to assume that none of them can lead to a vulnerability issue. New attacks and exploits [3,4] emerge every day, and it is impossible to prevent them all. However, in most cases, it is possible to find a solution to a newly discovered vulnerability and then fix it with a software or firmware update. A safe way to update the software or the firmware of a vehicle is to bring it to the nearest licensed workshop: clearly, this scenario must be avoided, since it can cause a serious disservice to the customer and also extra costs for the automotive OEM (Original Equipment Manufacturer). This is a serious problem that caught also the attention of the European Processor Initiative (EPI) project committee [5,6] and its partners such as BMW and Elektrobit. One solution is to update the software/firmware over the air (OTA), with the user that can manage the update in the same way as he/she does with a smartphone or a home PC. The basic idea is that inside each vehicle there is a special ECU, which is called gateway, that connects the outer world to all the vehicle’s ECUs. For example, the gateway provides for infotainment to the vehicle’s passengers or—in our case—an internet connection with the manufacturer to download the updates. There are many state-of-the-art solutions [1,7,8], which already implement OTA software update, and all of them focus on providing the authenticity and the integrity of the update. Confidentiality instead, is treated as an optional security feature. Unfortunately, the Intellectual Property (IP) of the update is not protected, since, in the case the update is sent to the vehicle without any encryption, the competitors can easily capture and analyze its content. This is not desirable, in particular, if the update contains innovative countermeasures to new attacks, or if it introduces new features for the vehicle. Another problem is that, even if the update is confidentially transmitted through the establishment of a secure channel (e.g., TLS), confidentiality is not guaranteed when the update is at rest. When a piece of data is “at rest”, it means that is not traveling through the internet, for example, data that are stored on a cloud server are at rest. This means that a manufacturer that uses secure channels to provide confidentiality to its updates cannot use a third-party untrusted server for storage and distribution, since, when an update is uploaded to such servers, it will not be encrypted. Furthermore, even if the manufacturer uses its own trusted cloud server to transmit the update to each vehicle, once the update arrives at the vehicle’s gateway, it can be still easily captured by someone who tampered with the gateway itself. This can happen since the gateway is the only ECU directly connected to the internet and, therefore, prone to cyber-attacks and more vulnerable to tampering than all of the other ECUs. Indeed, even in the Autosar Specification of Update and Configuration Management document, [9] it is specified that it is convenient to have a dedicated ECU, different from the gateway, in charge of managing the SW update of the vehicle. A solution to the "data at rest" problem is to asymmetrically encrypt the update itself (e.g., using RSA), so that only such a dedicated ECU (not even the gateway) is able to decrypt it. However, this approach can be costly, because it means that the manufacturer should encrypt the update as many times as there are vehicles to be updated. Attribute-Based Encryption (ABE) greatly reduces the cost of multiple-receiver end-to-end encryption, and solves the problem of update at rest, making it worthwhile and efficient to provide confidentiality. Using ABE, even if an adversary successfully tampers the vehicle’s gateway, then the update is still encrypted and signed with long-term keys in possession of the dedicated ECU, thus making the tampering useless. The only way that an adversary has to analyze a copy of the update is to tamper with either the dedicated ECU or the ECU that needs the update. The automotive industry is aware of such risks and, to contrast such issues, they developed some security strategies, such as the multi-layer security architecture [1]. Basically, the internal architecture of a vehicle is classified over different security levels that are based on the security requirements of the involved application, ranging from low-level security (e.g., infotainment) to high-level security (e.g., the brake system in an autonomous vehicle). Among other things, this strategy ensures that the ECUs that are not directly connected to the internet are hard to tamper with.

Despite that many high-quality works have been published during the years [10,11,12] presenting the feasibility of ABE on a wide range of devices, to the authors’ knowledge the literature has not tested the impact of the ABE on a real hardware automotive embedded platform. In this paper, our task is to demonstrate that ABE schemes, in general, are well supported by a platform that is very close to a real ECU mounted on a vehicle. In order to do this, we selected the CP-ABE by Bethencourt et al. [13], since is the one considered in the previously cited feasibility works. Showing that this scheme has little-to-no impact over the OTA process, we show that ABE is able to perform well over such a category of devices.

The contribution of this paper consists in: (i) show an ABE technique for OTA secure update of software/firmware that can be seamlessly integrated into state-of-the-art solutions; (ii) proving that ABE is compliant with the in-vehicle network organization in modern cars as well as with the computing capabilities of real automotive ECUs; and, (iii) provide an experimental evaluation of the ABE performances on a real automotive compliant platform, namely the Xilinx ZCU102 board. The rest of the paper is structured, as follows: in Section 2, we give some backgrounds and show the related works; in Section 3. we explain the setup of our performance evaluation; in Section 4, we show and discuss our results; and, finally, in Section 5, we conclude our paper.

## 2. Related Work

### 2.1. Attribute-Based Encryption

Attribute-Based Encryption (ABE) is a cryptographic technique that embeds an access control mechanism within the encrypted data. ABE describes the data and decrypting parties by means of attributes, and it regulates access to data with policies, which are Boolean formulas defined over these attributes. In ABE, an encrypting party (from now on, data producer) uses an encryption key, which is public and unique, whereas any decrypting party (from now on, data consumer) uses a decryption key, which is private and different for each of them. ABE has two main paradigms: Ciphertext-Policy Attribute-Based Encryption (CP-ABE) [13,14,15,16] and Key-Policy Attribute-Based Encryption (KP-ABE) [17,18,19,20]. In CP-ABE, each data consumer holds a list of attributes (attribute set), which is embedded in the decryption key. Each piece of data is described by one access policy, which is embedded in the ciphertext. An access policy can be seen as a tree, where the inner nodes are the logical operators “AND” and/or “OR” and the leaves are attributes. Four main algorithms are used in every CP-ABE scheme: Setup, KeyGen, Encrypt, and Decrypt.

- The Setup algorithm generates a master key and an encryption key;
- the KeyGen algorithm generates a decryption key, taking, as input, the master key and an an attribute set which describes the owner of the generated decryption key;
- the Encrypt algorithm generates a *ciphertext*, taking, as input, the encryption key, a message, and an access policy which describes the data being encrypted; and,
- the Decrypt algorithm takes as input a decryption key and a ciphertext, returning the decrypted message if and only if the attribute set satisfies the access policy.

KP-ABE shares the same algorithms with CP-ABE. However, in KP-ABE, the KeyGen algorithm takes, as input, an access policy rather than an attribute set, while the Encrypt algorithm takes as input an attribute set rather than a policy. ABE guarantees collusion resistance, which implies that two consumers cannot use their combined keys to access data that, singularly, neither of them can access. Furthermore, one of the main perks of ABE is that it allows for someone to encrypt a piece of information only once and mathematically enforcing on it an access control mechanism in such a way that only decryption keys with adequate access rights are able to decrypt them.

This means that, if a data producer wants to send a file to a number *n* of data producer using RSA, then the data producer has to encrypt such a file n times; instead, if the data producer uses ABE, it can encrypt such file only once. Therefore, a car company can encrypt an update with ABE, sign it, and upload it to some third-party cloud-storage servers that will then distribute such an update to all the target vehicles in a safe way, meaning that the transmission provides authenticity, integrity, and confidentiality of the received update. ABE is a secure design choice, even if the third-party cloud-storage server is not trusted. For our performance evaluation, we used the CP-ABE by Bethencourt et al. [13], since we think that a CP-ABE approach is the better solution for OEM, as the CP-ABE approach gives greater control to the data producer (i.e., the OEM) than a KP-ABE approach [21].

### 2.2. Over the Air Frameworks

The “Over the Air” update solution is the future of software and firmware update concerning the ECUs inside a vehicle. To the user, not having to bring the car to the nearest licensed workshop is a great relief, and it also can improve the chance that the update is actually installed. Moreover, the reported statistics show that automotive OTA can reduce warranty costs by a factor of 2 [22].

There are some state-of-the-art solutions that implement end-to-end encryption for OTA FW/SW update as *vConnect* [23]. Their solution is to establish a secure channel through an encrypted session between their servers and the vehicle’s gateway. This is dangerous because after the image download is completed, it can be considered “at rest” inside the gateway. The gateway is the most probable ECU to be compromised, since it is the only one that is directly connected to the internet. In contrast, if a company were to adopt the ABE OTA SW update technique shown in this paper, then the gateway should forward the downloaded encrypted and signed image to the Update and Configuration Manager (UCM). The UCM, according to the Autosar Adaptive specification document [9], can also run on a dedicated ECU different from the gateway and, therefore, more protected from external attacks.

In 2016, Karthik et al. [8] released Uptane, a Framework for software and firmware update over the air, created for securing ground vehicles. Uptane, optionally, allows for one to encrypt software images (i.e., software updates) while using symmetric, asymmetric, or digital envelope techniques. In this paper, we design a simple framework integrated with ABE to measure its impact on the OTA software update. Because ABE is, by all means, an asymmetric encryption scheme, we show that is possible to integrate it in a real and complex framework, therefore showing that ABE is a viable solution to provide confidentiality for the IP.

In 2018, Asokan et al. [7] proposed ASSURED, a framework for OTA firmware, which is based on Uptane [8]. In their work, they claim that assured reaches five objectives:

1. End-to-End authentication and integrity: the update must be signed by the manufacturer and verified by the device.
2. Update Authorization from Controller: only authorized devices can install the update.
3. Attestation of update installation: the device must provide proof of the update installation.
4. Protection of Code and secret key on device: the update must be stored and then installed in secure storage and isolated execution of critical code.
5. Minimal burden for the device.

However, ASSURED does not consider as an adversary an external entity that eavesdrops on the communication to retrieve the update’s code, or that retrieves it from a tampered gateway. Instead, in our work, in addition to the objectives that are achieved by ASSURED, we consider such an adversary and provide protection from it while using Attribute-Based Encryption.

In 2020, Ghosal et al. [24] proposed STRIDE, which is an OTA software update scheme for autonomous vehicles. In their work, the authors provide confidentiality to the software update by using the CP-ABE scheme that was proposed by Bethencourt et al. [13]. Furthermore, they provide an extensive performance evaluation by simulation through OMNeT++ [25]. However, they do not test the performance of the introduction of ABE on a real automotive platform, as we do in this paper with the Xilinx ZCU102 evaluation board. This gives us a realistic estimation of the performances. Moreover, the authors do not evaluate the performance of key revocation mechanisms, which cannot be neglected, as they are necessary for practical use in a real-world scenario.

Halder et al., published recently a survey [26] on secure over the air software updates in connected vehicles. In their work, the update’s confidentiality is a mandatory requirement, and they investigated and discussed many schemes. The covered techniques are, for example, OTA that is based singularly on: symmetric key; hash functions; blockchain; RSA and steganography; HSM; secure update frameworks; and, so on. However, in their work, an approach that is explicitly based on Attribute-Based Encryption has not been considered.

### 2.3. Testing Platforms and Automotive Hardware Background

The original Attribute-Based Encryption was proposed in 2005 by Sahai and Waters [27]. Since then, many researchers have proposed their own schemes, and they evaluated such schemes over various platforms. Moreover, many works have been written that discuss the feasibility of many ABE schemes (both KP-ABE and CP-ABE) on limited-resource devices, like smartphones [11] and IoT devices [10,12]. However, to the authors’ knowledge, the literature has yet to test the impact of ABE schemes on a real hardware automotive embedded platform. The main difference between traditional IoT devices (e.g., smartphones, sensors, Raspberry Pi, …) and automotive embedded platforms is that the latter feature different hardware. As the reader will see at the end of this section, automotive embedded platforms feature, among other things, multi-core processors and real-time processors, hardware that is not available in common IoT devices. Therefore, in this section, we explain the on-board network organization and computation capability of real automotive platforms, so that the proposed ABE technique is integrated into a representative automotive scenario. We reference emerging vehicle architectures, describing how it is designed, to show that previous works cannot be taken into consideration when arguing about the performances of ABE in the automotive domain.

In-vehicle networks are in a transition from legacy domain-based electronic architectures to zonal architectures. Domain-based architectures with many simple and separate ECUs and networks will be used for commodity automotive subsystems (e.g., break or steer control). Instead, for high-performance tasks (e.g., sensor fusion for obstacle detection, navigation, and trajectory planning), a small number of supercomputers is needed. Besides classic local interconnect and controller area networks, in emerging automotive platforms, the wireless V2X (vehicle to everything) connectivity is ensured by vehicular versions of WLAN (e.g., 802.11p technology) and of Cellular networks (e.g., C-V2X). This wide range of connectivity will highly increase the opportunity of SW OTA distributed dissemination [26]. However, such a wide range of connectivity solutions to the external world can be a liability, since it opens the vehicle to external cyber threats. This is perceived as a serious threat by the automotive companies, which tried to aggregate all of the connectivity capabilities over a single ECU, called the gateway. However, for critical applications, like the OTA SW/FW update, it is recommended to provide a dedicated ECU, different from the gateway, called the Update and Configuration Manager [9]. This ECU contains the cryptographic quantities that are needed for the OTA SW/FW update, such as public keys and private keys, for signature verification and decryption, respectively. Indeed, the recommended OTA update process inside the vehicle looks like this: (i) the gateway downloads the signed and encrypted update, and forwards it to the UCM; (ii) the UCM verifies the signature; (iii) the UCM decrypts the update; and finally, (iv) the UCM forwards the decrypted update to the ECU that needs it.

A key component of this new automotive networking architecture is the availability of more powerful ECUs than before. Differently from commodity ECUs, which are characterized by low-cost microcontrollers, powerful automotive ECU processors are typically equipped with (i) interfaces towards Ethernet physical layer and switches, (ii) application processors like those of the Cortex-A family with AArch64 64-bit instruction set, and (iii) Hardware Security Engine (HSE) for secure boot and accelerated security services. Referring to point (iii), in particular, the UCM should be a “security level IV” ECU, as specified by the de-facto standard on vehicular security hardware, the *EVITA* project [28]. In terms of intra-vehicle connectivity, the S32G chip sustains several network protocols, like Ethernet and CAN. Those characteristics allows for the UCM to easily perform many cryptographic operations and also to be connected with every commodity ECUs, which needs support for the OTA SW/FW update.

The reader should be aware that such resourceful ECUs are not future developments, but they are already being used. The same concept of integrating multi-core Cortex-A processors in automotive platforms is also followed by supercomputer platforms, like the Renesas H3 heterogeneous System-on-Chip. It integrates four Cortex-A72 and four Cortex-A53 cores, supervised by a dual lock-step Cortex-R7 real-time microcontroller and a rich set of networking interfaces. To this aim, the European Processor Initiative [6] is developing a High-Performance heterogeneous processor that integrates multiple ARM cores with AArch 64-bit architecture with SVE (Scalable Vector Extension) plus co-processor tiles for embedded FPGA, massively parallel processor array (MPPA) and RISC-V based stencil and neurostream accelerators (STX). In order to assess the easy integration of ABE as an additional feature, this work provides performance evaluation on a real automotive compliant board. To this aim, the ZCU102 from Xilinx has been selected as a versatile prototyping platform, which is representative of both automotive powerful ECUs processors, and of scaled versions of heterogeneous supercomputers. The ZCU102 hosts a Zynq UltraScale+ MPSoC chip with quad Arm Cortex^®^-A53 cores with Arm Neon™ technology plus dual-core Cortex-R5F real-time processors, a Mali™-400 MP2 graphics processing unit. The FPGA resources of the ZCU102 allow for further accelerators integration. ZCU102 also provides a rich set of connectivity interfaces. From a SW development point of view, the ZCU102 sustains a Linux-like OS (PetaLinux) and an integrated design environment (VITIS) in order to develop both HW and SW parts. It is to be noted that the Xilinx Zynq UltraScale+ MPSoC platform is not only a rapid prototyping tool, but it can be also used as a product, being recently adopted by Continental for its four-dimensional (4D) automotive radar [29].

## 3. Methods

**Figure 1:**
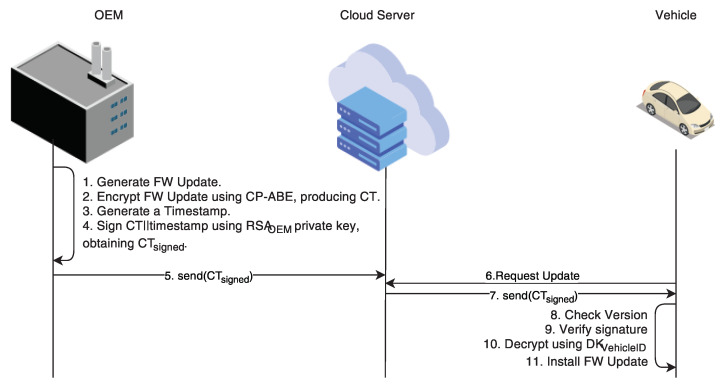
Use-case scenario of firmware over-the-air update using Ciphertext-Policy Attribute-Based Encryption (CP-ABE).

We designed the following experiments in order to evaluate the impact that the introduction of ABE has on a vehicle’s performance. As ABE scheme for the experiments, we choose the CP-ABE by Bethencourt et al. [13]. By default, CP-ABE encryption is performed using the digital envelope technique. This means that the CP-ABE ciphertext protects a symmetric key, which is used to symmetrically encrypt the actual message. In our case, it is the Advanced Encryption Standard (AES). Throughout this paper, we will consider CP-ABE encryption and CP-ABE decryption as always using the digital envelope technique. Our scenario is composed of many vehicles, a manufacturer, and an honest-but-curious cloud server. The manufacturer possesses the CP-ABE master key, the CP-ABE encryption key, a pair of RSA keys, and knows each vehicle’s RSA public key. The manufacturer is in charge of generating all of the cryptographic keys needed in the system. We assume that each vehicle has an ECU dedicated to the OTA update called Update and Configuration Manager (UCM), as specified in the Autosar specification document [9]. This ECU is not connected directly to the internet, although it is connected to the gateway, and to each ECU that supports the OTA update functionality. Each vehicle possesses: (i) a CP-ABE decryption key, which describes the vehicle’s components and characteristics; (ii) a pair of RSA keys; and, (iii) the manufacturer’s RSA public key. These vehicle-related cryptographic keys are installed in the ECU that implements the UCM [9] by the OEM at the time of its construction. The manufacturer is in charge to produce the software update, encrypts it with CP-ABE, signs it—along with a version number—using RSA, and stores the signed and encrypted update on the cloud server. The cloud server sends the signed and encrypted update to any vehicle that requests it. Upon reception of the software update, the gateway forwards the message to the UCM, which first verifies the manufacturer signature, and then it decrypts the CP-ABE ciphertext. Finally, the UCM forwards the software update to the intended ECU, which installs it as soon as the user gives his/her consent. Figure 1 depicts the use case and the interactions.

**Figure 2:**
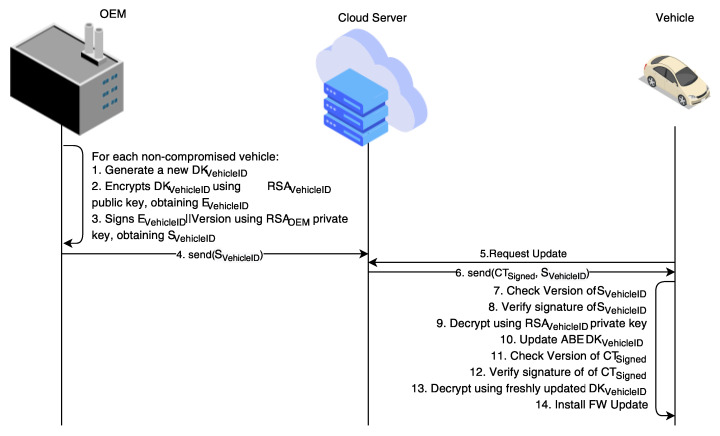
CP-ABE decryption key distribution in case of key compromised.

Furthermore, in the case one or many decryption keys are compromised, the manufacturer also provides new keys to the non-compromised vehicles. To do so, the manufacturer generates a new CP-ABE decryption key for each non-compromised vehicle, encrypt said key using the vehicle’s RSA public key, and signs the ciphertext while using its RSA private key. Subsequently, the manufacturer stores the encrypted and signed decryption key (from now on, the key update) in the cloud server. If a new decryption key has been released for a vehicle, when such a vehicle requests a software update, the cloud server also sends to it the key update. In this case, the UCM first verifies the signature on the key update, and then retrieves the new CP-ABE decryption key while using RSA decryption. Finally, the UCM verifies the signature on the software update and decrypts it using the new CP-ABE decryption key. Figure 2 depicts such interactions.

The reader may argue that this revocation mechanism is inefficient. However our example is a worst-case scenario: if the impact of such a naive mechanism is limited on our test platform, then this means that more advanced and efficient revocation mechanisms will be well supported too.

### Attacker Model

With reference to Figure 2, which represents the most complex scenario treated in this paper, we now define the attacker model: its capabilities, its motivation, its objectives, and how it would like to achieve them.

We assume that the OEM, the UCM, and all the vehicle’s ECUs (except the gateway) are trusted, whereas the Cloud Server and gateway are considered to be untrusted. We think that this is a good assumption to make, since the gateway inside a vehicle is the only ECU that is directly connected to the internet and, therefore, it is more exposed to external attacks than the other ECUs.

We now analyze the considered threats: a passive attacker and an active attacker. A passive attacker is able to intercept every message that is sent over the internet, both between the OEM and Cloud Server and between the Cloud Server and vehicle. The objectives of a passive attacker are two: (p_i) to capture and decrypt an ABE ciphertext, obtaining an update; and, (p_ii) to capture and decrypt an RSA ciphertext to retrieve an ABE decryption key. However, objective (p_i) and (p_ii) cannot be achieved, since it would mean that the attacker is able to break the schemes in [13] and [30], respectively.

The active attacker, instead, is able to gain access to and/or control of the Cloud Server and/or the gateway. This can be done by leveraging one of the many vulnerabilities that have been discovered over the years [3,4,31]. For example, an active adversary is able to install a spyware on the gateway (or on the cloud server), so that each and every piece of information that is managed and manipulated by it is forwarded to the attacker.

The objectives of an active attacker are: (a_i) to force a vehicle to install a malicious SW update; (a_ii) to decrypt an ABE ciphertext; and, (a_iii) to acquire a decryption key and a private RSA key from a vehicle.

Objective (a_i) cannot be achieved without forging a valid signature applied to the SW update by the OEM, therefore breaking the RSA signature scheme. The attacker can pursue objective (a_ii) by gaining control of the cloud server, or by gaining control of the gateway. In both cases, however, the attacker cannot achieve such an objective since neither the Cloud Server nor the gateway possesses any decryption key. If in some way, the attacker retrieves a vehicle’s CP-ABE decryption key and the RSA private key—achieving (a_iii)—it will be capable of decrypting any ciphertext that such ABE key complies with, and it will be capable of retrieving and decrypting the key update made for said vehicle. This attack is effective until the OEM performs a revocation for the key. When the OEM does this, it removes the associated RSA public key from its database of public keys, and it stops generating key updates for the compromised vehicle. The methods through which the OEM learns about the attacks and, therefore, it is able to issue a revocation, and fall out of the scope of this paper.

## 4. Performance Evaluation

In this section, we briefly explain how we recreated the scenario, showing the software and hardware that we used.

### 4.1. Experimental Setup

**Figure 3:**
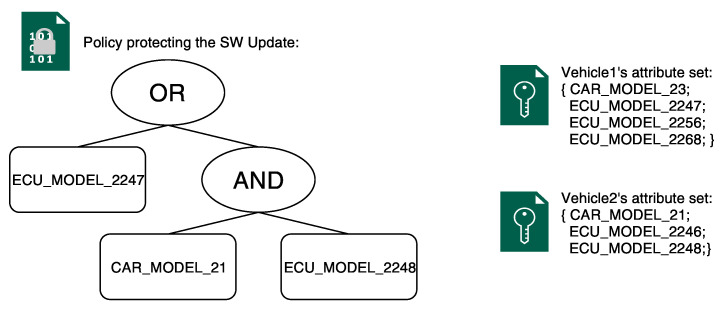
The policy and the two attribute sets used for the experiments.

We designed a client-server application that reflects the interaction between the cloud server and the vehicles. We used C as the programming language and OpenSSL, libswabe, the CP-ABE toolkit [32], GMP, and the Pairing Based Cryptography (PBC) as libraries. The objective is to measure the time that passed from the moment an update is requested to the moment it is installed. Ultimately, we show that CP-ABE has little-to-no impact on the performance while providing a fundamental feature. We investigated three different scenarios: (i) NO CP-ABE; (ii) only CP-ABE encryption; and, (iii) CP-ABE encryption + key update. In the first scenario, when the vehicle requests an update, the cloud sends to the vehicle the update in the clear along with the associated version, all being signed by the OEM. This scenario will be our reference for the CP-ABE performance evaluation. In the second scenario, Figure 1 depicts the interaction between the cloud server and the vehicle. The cloud server stores the SW update encrypted with CP-ABE, along with the update’s version, all being signed by the OEM. In the third scenario, the interaction between the cloud server and the vehicle is similar to the one in the second scenario. However, every once in a while, in addition to the SW update encrypted with CP-ABE, the cloud server will also send to the vehicle a new CP-ABE decryption key as depicted in Figure 2. For scenario 2 and 3, we used a single policy to encrypt the software update, and two different attribute sets to represent two different vehicles (Vehicle1 and Vehicle2) that are both able to satisfy the policy. Figure 3 depicts the policy and attribute sets. The policy reads as: “a vehicle can access the data if and only if it has the *ECU_MODEL_2247* **OR** it is both a *CAR_MODEL_21* **AND** it has the *ECU_MODEL_2248*”. The attribute set of Vehicle1 is composed of four different attributes and it reads as follow: “Vehicle1 is a *CAR_MODEL_23* and it has *ECU_MODEL_2247*, *ECU_MODEL_2256*, and *ECU_MODEL_2268*”; the attribute set of Vehicle2 is composed of three different attributes and it reads, as follow: “Vehicle2 is a *CAR_MODEL_21* and it has *ECU_MODEL_2246*, and *ECU_MODEL_2248*”. Vehicle1 is able to decrypt the ciphertext, because it has the *ECU_MODEL_2247* attribute, while Vehicle2 is able to decrypt the ciphertext, because it has both the attributes *CAR_MODEL_21* and *ECU_MODEL_2248*.

We run the client (which simulates the vehicle) on a Xilinx ZCU102 evaluation board that is equipped with a Zynq UltraScale+ MPSoC chip which features, as already discussed before, a quad Arm Cortex^®^-A53 cores with Arm Neon™ technology plus dual-core Cortex-R5F real-time processors, a Mali™-400 MP2 graphics processing unit, and four SLFP+ interfaces for Ethernet, 6 16.3 Gb/s GTH transceivers, and 64 user-defined differential I/O signals, 600 system logic cells, 32 Mb of memory, 2500 DSP slices.

For each scenario, we evaluated the performances of the ZCU102 board over 5000 iterations, with a confidence interval of 95%. For the Elliptic Curve Cryptography operations, we used Type A internals of PBC library with group order of 160 bits, element size of 512 bits, and an embedding degree k=2, which gives an 80-bit security level. In order to chose the revocation rate, we based our analysis on the frequency of update in Tesla vehicles [33]. From their websites, we can see that, from January 2020 to November 2020, 122 updates have been released, meaning that—on average—more than 11 updates are released each month. Therefore, we evaluated four different revocation rate, roughly from weekly to monthly: once every two updates, once every three updates, once every six updates, and once every 12 updates.

### 4.2. Results

**Figure 4:**
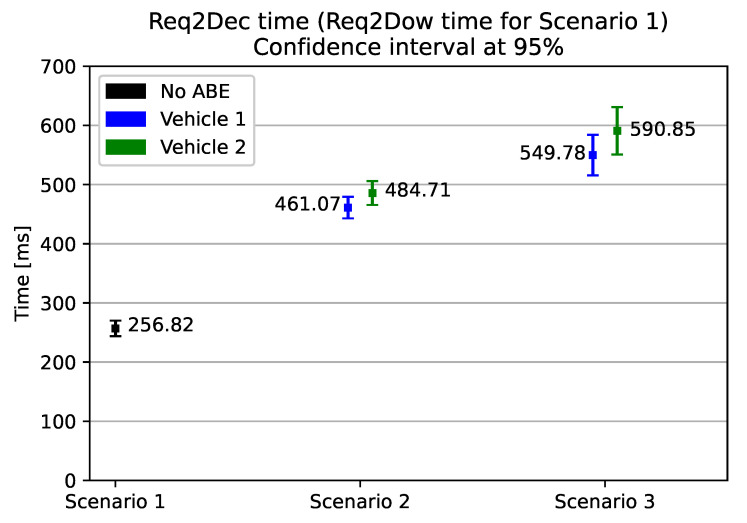
Elapsed time from the update request to the moment just before the installation. The considered revocation frequency in Scenario 3 is once every six updates.

**Figure 5:**
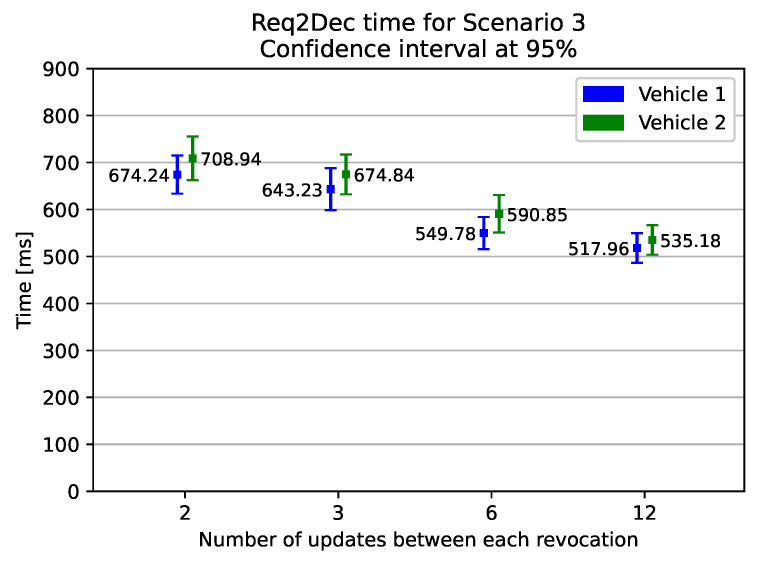
Elapsed time from the update request to the moment just before the installation in scenario 3, varying the revocation frequency.

We show, in Figure 4, the results of our experiment. The results of scenario 1’s evaluation show us that the download time and the verification of the RSA signature takes about 256 ms. The introduction of only CP-ABE decryption in scenario 2 increases by 200–230 ms the time elapsed from the request of the update to the starting of the installation. This increase in time is due to CP-ABE decryption and the CP-ABE ciphertext overhead download. From the graph, we can see that Vehicle2 spends, on average, about 24 ms more than Vehicle1. Indeed, in order to decrypt the CP-ABE ciphertext, in Vehicle2 the attribute used to decrypt the policies are 2 (i.e., *CAR_MODEL_21* and *ECU_MODEL_2248*), whereas Vehicle1 only has 1 (i.e., *ECU_MODEL_2247*). Finally, in scenario 3, we see that the additional seldom retrieval of a new decryption key costs, on average, 90–105 ms, in the case of a revocation frequency of once every six updates, which translates to a revocation every 15 days. Moreover, Figure 5 shows that, for a wide range of revocation frequencies, the impact of CP-ABE decryption and key update is limited. Even at the higher measured frequency—once every two updates, or once every five days—the download, key update, and the decryption processes are all performed in just under 675–710 ms. If the revocation frequency drops to once every 12 updates (roughly, once a month), the entire process takes between 518–535 ms.

**Figure 6:**
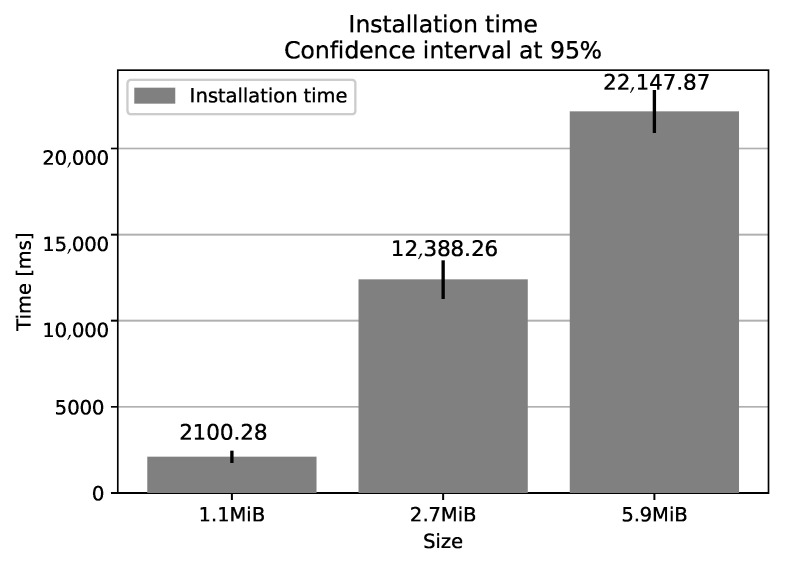
A comparison of the installation times of various SW’s size.

Analyzing the results of the time spent in scenarios 2 and 3 as compared to the time spent in scenario 1, it seems that CP-ABE has a non-negligible impact on the OTA SW update process. However, when we compare these results to the time actually spent on the installation of the SW, we can see that the time increase due to CP-ABE is negligible. Indeed, we also investigated the size of a software update in the automotive scenario. We found out that, typically, an update’s size for a Tesla “Model 3” is about 100 MB [34]. In order to replicate the installation process, we chose to install installation packages of different sizes on the ZCU102. Namely, we measured the installation time for programs with sizes of ∼6.9 KiB, ∼2.7 MiB, ∼5.9 MiB, as shown in Figure 6. We did not perform tests of greater SW size for two reasons: (i) we had difficulties to find programs with the size around 100 MB; and, (ii) even with such small sizes, the installation times are already orders of magnitude greater than decryption and download time. Figure 6 shows that the software of size ∼6.9 KiB, ∼2.7 MiB, ∼5.9 MiB took, on average, 2100 ms, 12,388 ms, and 22,148 ms, respectively.

**Figure 7:**
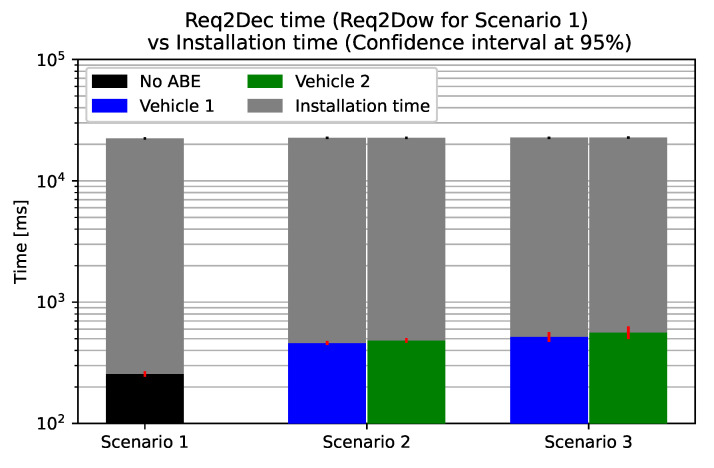
A comparison of the total time taken from the update request to the end of SW installation. The considered SW size is 5.9 MiB, and the considered revocation frequency is once every 6 updates.

In Figure 7, we can see how much time it takes for each scenario from the update request to the end of the update installation. We had to use the logarithmic scale in order to see the CP-ABE impact, since, when considering the installation time, the three scenarios are practically equivalent. In fact, the average time spent on the 5.9 MiB SW update is 22,148 ms with a 95% confidence interval of ±247 ms. This means that the SW installation time is two orders of magnitude greater than all of the previous time computed in the three presented scenarios. Therefore, we can conclude that the time impact of CP-ABE is negligible when also considering that we measured the installation times on SW images that are way smaller of the ones deployed in reality.

**Figure 8:**
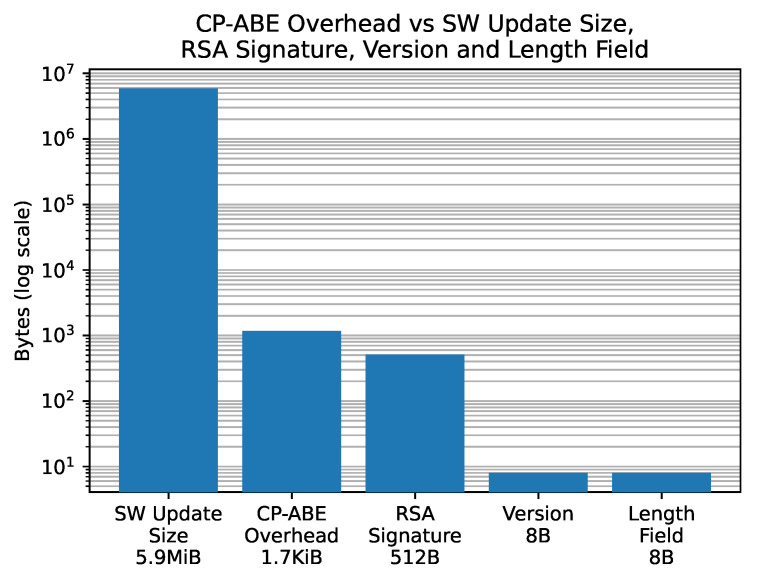
The size of each field inside the update message that the cloud sends to the vehicle.

Furthermore, we also considered the impact of CP-ABE in terms of message size. Figure 8 shows the size of the single components of the update message that the cloud sends to the vehicle. The main three fields of such a message are: (i) the symmetrically encrypted SW update (5.9 MiB); (ii) the CP-ABE ciphertext containing the symmetric key; and, (iii) the RSA signature of the OEM. When compared to the RSA signature, the CP-ABE ciphertext amounts to over three times the size. However, compared to the actual software update size, the impact of the CP-ABE ciphertext is so negligible that, in order to show them both in the same graph, we have to use a logarithmic scale.

When considering that the OTA SW update operation is not a time-critical task and, when considering that, in any case, the dominant time cost is the SW installation, we think that the adoption of CP-ABE also in real-life application will be a great addition for the security of our vehicles.

## 5. Conclusions and Future Works

This paper has shown that the Attribute-Based Encryption technique improves the security for over the air update functionalities in an automotive scenario. Particularly, Attribute-Based-Encryption (ABE) provides confidentiality to the software/firmware update done Over The Air and also for data at rest. This is a feature that is missing in alternative solutions that are available at the state of the art. Furthermore, we tested a naive key revocation mechanism, which is another missing feature in the state-of-the-art systems. The paper has demonstrated that ABE can be seamlessly integrated into the existing solutions regarding the OTA update and, more broadly, it complies with automotive standards in terms of architecture and documentations. Furthermore, the overhead of the ABE integration in terms of computation time and storage is negligible w.r.t. the other tasks that are involved in an OTA software update, like the installation. These results show that security can be enhanced at a minimum cost. Because the University of Pisa is an official partner of the EPI project [5] within the H2020 research program, we plan to work and cooperate with other partners, such as BMW and Elektrobit, in order to integrate our work in their already existing systems. In particular, we plan to port the OTA CP-ABE technique to be AUTOSAR-adaptive [35] compliant.
